# Supplementary material for: High dispersal levels and lake warming are emergent drivers of cyanobacterial community assembly in peri-Alpine lakes
Source: Sci Rep. 2019 May 14;9:7366. doi: 10.1038/s41598-019-43814-2 (PMC6517590; doi:10.1038/s41598-019-43814-2)
Supplement: Supplementary file 1 — supplementary online information [file 41598_2019_43814_MOESM1_ESM.docx]

**Supplementary Information**

**High dispersal levels and lake warming are emergent drivers of cyanobacterial community assembly in peri-Alpine lakes**

Authors: Marie-Eve Monchamp^1,2^, Piet Spaak ^1,2^, Francesco Pomati ^1,2,#^

^1^ Eawag, Swiss Federal Institute of Aquatic Science and Technology, Department of Aquatic Ecology, 8600 Dübendorf, Switzerland;

^2^ Swiss Federal Institute of Technology (ETH) Zürich, Institute of Integrative Biology, 8092 Zürich, Switzerland.

# Corresponding author:

Eawag, Department of Aquatic Ecology

Überlandstrasse 133, 8600 Dübendorf, Switzerland.

Email: [francesco.pomati@eawag.ch](mailto:francesco.pomati@eawag.ch)

Phone: +41 58 765 5410

**Supplementary methods**

**Distance-decay relationship**

The *GeoDistanceInMetresMatrix* function for R (<https://eurekastatistics.com/calculating-a-distance-matrix-for-geographic-points-using-r/>) was used to derive a matrix of geographical distances between lakes (Table S2).

*## GeoDistanceInMetresMatrix* function:

function(df.geopoints){

# Returns a matrix (M) of distances between geographic points.

# M[i,j] = M[j,i] = Distance between (df.geopoints$lat[i], df.geopoints$lon[i]) and

# (df.geopoints$lat[j], df.geopoints$lon[j]).

# The row and column names are given by df.geopoints$name.

GeoDistanceInMetres <- function(g1, g2){

# Returns a vector of distances. (But if g1$index > g2$index, returns zero.)

# The 1st value in the returned vector is the distance between g1[[1]] and g2[[1]].

# The 2nd value in the returned vector is the distance between g1[[2]] and g2[[2]]. Etc.

# Each g1[[x]] or g2[[x]] must be a list with named elements "index", "lat" and "lon".

# E.g. g1 <- list(list("index"=1, "lat"=12.1, "lon"=10.1), list("index"=3, "lat"=12.1, "lon"=13.2))

DistM <- function(g1, g2){

require("Imap")

return(ifelse(g1$index > g2$index, 0, gdist(lat.1=g1$lat, lon.1=g1$lon, lat.2=g2$lat, lon.2=g2$lon, units="m")))

}

return(mapply(DistM, g1, g2))

}

n.geopoints <- nrow(df.geopoints)

# The index column is used to ensure we only do calculations for the upper triangle of points

df.geopoints$index <- 1:n.geopoints

# Create a list of lists

list.geopoints <- by(df.geopoints[,c("index", "lat", "lon")], 1:n.geopoints, function(x){return(list(x))})

# Get a matrix of distances (in metres)

mat.distances <- ReplaceLowerOrUpperTriangle(outer(list.geopoints, list.geopoints, GeoDistanceInMetres), "lower")

# Set the row and column names

rownames(mat.distances) <- df.geopoints$name

colnames(mat.distances) <- df.geopoints$name

return(mat.distances)

}## End

**Supplementary Table S1.** List of primer sequences and primer tag sequences used in this study. The primers amplify a ~400bp-long fragment part of the V3-V4 region of the 16S cyanobacterial genes ^1–3^.

| **Name forward primer** | **Tag forward primer** | **Forward primer sequence** | **Name reverse primer** | **Tag reverse primer** | **Reverse primer sequence** |
| --- | --- | --- | --- | --- | --- |
| CYA359F -1 | NNAGAGCTAC | GGGGAATYTTCCGCAATGGG | CYA784R -1 | NNTATCCTCC | GACTACWGGGGTATCTAATCCC |
| CYA359F -2 | NNCGCATAGA | GGGGAATYTTCCGCAATGGG | CYA784R -2 | NNAACACCGT | GACTACWGGGGTATCTAATCCC |
| CYA359F -3 | NNGGTAGTTCT | GGGGAATYTTCCGCAATGGG | CYA784R -3 | NNTTACCGCT | GACTACWGGGGTATCTAATCCC |
| CYA359F -4 | NNCGTAAGTC | GGGGAATYTTCCGCAATGGG | CYA784R -4 | NNTGAGATGC | GACTACWGGGGTATCTAATCCC |
| CYA359F -5 | NNAACAGCTC | GGGGAATYTTCCGCAATGGG | CYA784R -5 | NNNGTGCAACT | GACTACWGGGGTATCTAATCCC |
| CYA359F -6 | NNAACTGTCC | GGGGAATYTTCCGCAATGGG | CYA784R -6 | NNNTGAGCCTA | GACTACWGGGGTATCTAATCCC |
| CYA359F -7 | NNNTCCTGGTA | GGGGAATYTTCCGCAATGGG | CYA784R -7 | NNNATGGAGGT | GACTACWGGGGTATCTAATCCC |
| CYA359F -8 | NNNCATCCAGT | GGGGAATYTTCCGCAATGGG | CYA784R -8 | NNNCTGAGTCT | GACTACWGGGGTATCTAATCCC |
| CYA359F -9 | NNNCAACCTCA | GGGGAATYTTCCGCAATGGG | CYA784R -9 | NNNGAGGTGAA | GACTACWGGGGTATCTAATCCC |
| CYA359F -10 | NNNTGCTTGTC | GGGGAATYTTCCGCAATGGG | CYA784R -10 | NNNGGCATGTA | GACTACWGGGGTATCTAATCCC |
| CYA359F -11 | NNNCGTGATAC | GGGGAATYTTCCGCAATGGG |  |  |  |
| CYA359F -12 | NNNCTTCTTCC | GGGGAATYTTCCGCAATGGG |  |  |  |

**Supplementary Table S2.** Pairwise geographic distances across lakes (kilometres).

|  | Lugano | Baldeggersee | Constance | Hallwilersee | Pusiano | Maggiore | Annecy | Geneva | Greifensee | Zurich |
| --- | --- | --- | --- | --- | --- | --- | --- | --- | --- | --- |
| Lugano | 0 |  |  |  |  |  |  |  |  |  |
| Baldeggersee | 147 | 0 |  |  |  |  |  |  |  |  |
| Constance | 183 | 101 | 0 |  |  |  |  |  |  |  |
| Hallwilersee | 157 | 10 | 101 | 0 |  |  |  |  |  |  |
| Pusiano | 22 | 169 | 200 | 179 | 0 |  |  |  |  |  |
| Maggiore | 26 | 142 | 192 | 151 | 41 | 0 |  |  |  |  |
| Annecy | 217 | 220 | 317 | 222 | 229 | 191 | 0 |  |  |  |
| Geneva | 195 | 156 | 256 | 157 | 212 | 171 | 72 | 0 |  |  |
| Greifensee | 155 | 36 | 65 | 37 | 175 | 156 | 255 | 192 | 0 |  |
| Zurich | 144 | 32 | 70 | 36 | 165 | 145 | 248 | 187 | 11 | 0 |

*Table adapted from* ^4^

**Supplementary Figures**

**Supplementary Figure S1.** Map of the European peri-Alpine region showing the location of the lakes sampled across the Northern and Southern Alpine Plateau. Detailed information on the morphology and physical-chemical characteristics of the ten lakes is available in ^3^. The map was created with ESRI ArcMap version 10.3.1 using Swisstopo data (adapted from ^3^).

**Supplementary Figure S2.** **Temporal decay of phylogenetic similarity across all lakes.** The beta-MNTD (mean-nearest-taxon-distance) values are plotted against a natural log-transformed temporal gradient (years). A significant increase in the mean nearest taxon distance with time is observed in communities of lakes Lugano, Hallwilersee, Maggiore, and Zurich (*p* > 0.05) (Lake Annecy; insufficient observations).

**Supplementary Figure S3.** **Temporal decay of phylogenetic similarity across all lakes.** The beta-MPD (mean-pairwise-distance) values are plotted against a natural log-transformed temporal gradient (years). A significant increase in the mean pairwise distance with time is observed in communities of lakes Hallwilersee, Pusiano, Maggiore, and Zurich (*p* > 0.05) (Lake Annecy; insufficient observations).

**Supplementary Figure S4.** **Temporal decay of phylogenetic similarity across all lakes.** The UniFrac distances are plotted against a natural log-transformed temporal gradient (years). A significant decay of phylogenetic similarity with time is observed in all lakes, with the exception of lake Geneva (*p* > 0.05) and lake Annecy (insufficient observations).

**Supplementary Figure S5. Cyanobacterial UniFrac** **beta-diversity over environmental gradients.** UniFrac pairwise similarities across lake communities are plotted against envirnmental distances (euclidean) based on **a)** total phosphorus concentrations in the water column, **b)** mean annual air temperatures, **c)** NO_3_^-^. concentrations in the water column, and **d)** Maximal water stability index. Significant relationships at the *p* ≤ 0.05 level are shown (TP; *p* = 0.005, adjusted R^2^ = 0.0120, DF = 559, Air temperature; *p* = 1.497^e-15^, Adjusted R^2^ = 0.0642, DF = 944). Samples used in the regression analysis are the forty-four non-random assemblages identified in Fig. 2 (i.e., communities showing significant phylogenetic structure based on SES_MNTD_ deviation from the null model expectation).

**Supplementary Figure S6. Cyanobacterial MPD beta-diversity over environmental gradients.** Mean pairwise distance (MPD) metric used for calculating beta-diversity across lake communities is plotted against envirnmental distances (euclidean) based on **a)** total phosphorus concentrations in the water column, **b)** mean annual air temperatures, **c)** NO_3_^-^. concentrations in the water column, and **d)** maximal annual water stability index. Significant relationships at the *p* ≤ 0.05 level are shown (Water stability index; *p* = 3.05^e-08^, adjusted R^2^ = 0.1333, DF = 208). Samples used in the regression analysis are the forty-four non-random assemblages identified in Fig. 2 (i.e., communities showing significant phylogenetic structure based on SES_MNTD_ deviation from the null model expectation).

**Supplementary Figure S7. Cyanobacterial beta-diversity over ammonia gradient.** a) Phylogenetic similarity based on **UniFrac**, b) beta-diversity based on mean-pairwise-distance (**MPD**), and c) beta-diversity based on mean-nearest-taxon-distance (**MNTD**) between samples plotted against environmental gradients (euclidean distances) based on NH_4_^+^ concentrations in the water column. Samples used in the regression analysis are the forty-four non-random assemblages identified in Fig. 2 (i.e., communities showing significant phylogenetic structure based on SES_MNTD_ deviation from the null model expectation).

**References**

1. Nübel, U., Garcia-pichel, F., Muyzer, G., Nu, U. & Muyzer, G. PCR primers to amplify 16S rRNA genes from cyanobacteria. *Appl. Environ. Microbiol.* **63,** 3327–3332 (1997).

2. DeSantis, T. Z. *et al.* Greengenes, a chimera-checked 16S rRNA gene database and workbench compatible with ARB. *Appl. Environ. Microbiol.* **72,** 5069–72 (2006).

3. Monchamp, M.-E. *et al.* Homogenization of lake cyanobacterial communities over a century of climate change and eutrophication. *Nat. Ecol. Evol.* **2,** 317–324 (2018).

4. Monchamp, M.-E., Spaak, P. & Pomati, F. Long Term Diversity and Distribution of Non-photosynthetic Cyanobacteria in Peri-Alpine Lakes. *Front. Microbiol.* **9,** 3344 (2019).
